# The Genetic Association of Polycystic Ovary Syndrome and the Risk of Endometrial Cancer: A Mendelian Randomization Study

**DOI:** 10.3389/fendo.2021.756137

**Published:** 2021-11-05

**Authors:** Hanxiao Chen, Yaoyao Zhang, Shangwei Li, Yuanzhi Tao, Rui Gao, Wenming Xu, Yihong Yang, Kemin Cheng, Yan Wang, Lang Qin

**Affiliations:** ^1^ Reproductive Centre, Department of Obstetrics and Gynaecology, West China Second University Hospital, Sichuan University, Chengdu, China; ^2^ West China School of Medicine, Sichuan University, Chengdu, China; ^3^ Key Laboratory of Birth Defects and Related Diseases of Women and Children of the Ministry of Education, West China Second University Hospital, Sichuan University, Chengdu, China; ^4^ Sichuan University-The Chinese University of Hong Kong (SCU–CUHK) Joint Laboratory for Reproductive Medicine, West China Second University Hospital, Sichuan University, Chengdu, Sichuan, China; ^5^ Outpatient Department, West China Second University Hospital, Sichuan University, Chengdu, China; ^6^ Department of Obstetrics and Gynaecology, Sichuan Academy of Medical Sciences & Sichuan Provincial People’s Hospital, Chengdu, China

**Keywords:** polycystic ovary syndrome, endometrial cancer, Mendelian randomization, GWAS, SNP

## Abstract

The association between polycystic ovary syndrome (PCOS) and endometrial cancer remains unclear. We aimed to investigate the causal association between genetically predicted PCOS and endometrial cancer risk in two ethnic groups through a two-sample Mendelian randomization (MR) approach. Our study includes 13 single nucleotide polymorphisms (SNPs) as instrumental variables (IVs) for PCOS in Europeans, and another 13 SNPs are used as IVs for PCOS in Asians. Outcome data were obtained from the largest published meta-GWAS of European ancestry to date, as well as from the BioBank Japan Project of Asian ancestry. Our study demonstrates that genetically predicted PCOS is not causally associated with the risk of overall endometrial cancer in either Europeans or Asians (odds ratio (OR) = 0.93, 95% confidence interval (CI) = 0.85–1.01, p = 0.09 and OR = 0.98, 95% CI 0.84–1.13, p = 0.75, respectively). Subgroup analyses according to histotype further illustrate that PCOS might not be associated with the risk of either endometrioid endometrial cancer or non-endometrioid endometrial cancer in European ancestry. No pleiotropy is found in our study, and a sensitivity analysis shows similar results. Our results indicate that genetically predicted PCOS might not be associated with the risk of endometrial cancer.

## Introduction

Polycystic ovary syndrome (PCOS) is one of the most prevalent reproductive endocrine disorders affecting five to 10 percent of reproductive-aged women around the world, and it greatly influences patient quality of life, fertility and long-term health (1, 2). PCOS is an unexplained heterogeneous clinical syndrome that might be caused by a combination of genetic and environmental factors. Due to the heterogeneity of PCOS, it is clinically diagnosed by multiple diverse diagnostic criteria according to the Androgen Excess and PCOS Society (3), the National Institutes of Health/National Institute of Child Health and Human Disease (NIH/NICHD) (4) and the European Society for Human Reproduction and Embryology/American Society for Reproductive Medicine (ESHRE/ASRM) (Rotterdam criteria) (5). Although there is no consensus on the clinical definition of PCOS, it is well accepted that hyperandrogenism and oligo-ovulation are the main characteristics of PCOS (6). Women with PCOS are at higher risk for comorbidities, including obesity, hyperinsulinemia, insulin resistance (IR), metabolic disorders, infertility, endothelial dysfunction, cardiovascular disorders and the development of cancer. Previous studies have found that PCOS was associated with an increased risk of cancer in the endometrium, ovaries, endocrine glands, pancreas, kidneys and skeletal and hematopoietic system (7, 8).

Endometrial cancer, rising in both incidence and associated mortality, is a common gynecological tumor (9). It is well known that a family history of endometrial cancer, aging, obesity, unopposed estrogen exposure (from hormone replacement therapy and tamoxifen use, for example), never having given birth, early age at menarche and late-onset menopause are risk factors of endometrial cancer (10). The results, however, of epidemiologic studies regarding the association between PCOS and endometrial cancer remain inconclusive (11). Most of the prior studies are observational studies, the findings of which could have possibly been affected by confounding factors and reverse causality due to the study design. For instance, many studies failed to control body mass index (BMI), which is an important potential confounder correlated with both PCOS and endometrial cancer risk (1, 12), leading to inaccurate estimates of their association. Moreover, endometrial cancer is most commonly classified into two subtypes according to clinical and histological characteristics: Type I endometrial cancer, which is usually estrogen dependent, is a uterine endometrioid carcinoma. Type II endometrial cancer, which is estrogen independent, has a non-endometrioid histology including serous carcinoma, carcinosarcoma, clear cell carcinoma, mucinous carcinoma and mixed histology types (13). Different subtypes of endometrial cancer may have different etiological risk factors, but none of the previous studies have stratified their outcomes. Consequently, without further stratification, the measurement of the association between PCOS and the risk of endometrial cancer might be unclear and inaccurate.

Mendelian randomization (MR), compared to conventional observational studies, can provide relatively strong and accurate evidence (14). MR analysis is less susceptible to potential environmental or social confounders, as well as reverse causality, because it uses genetic variants that are strongly and solely related to exposure as instrumental variables (IVs) to establish the association between exposure and outcomes. Furthermore, a two-sample MR study, an MR analysis based on data from two independent genome-wide association studies (GWASs), can provide strong evidence of the casual association resulting from their large sample sizes and increased statistical power (15). Several studies using two-sample MR analysis have found the potential relationship between PCOS and gynecologic neoplasms. One study demonstrated that PCOS was causally associated with a reduced risk of invasive ovarian cancer (16). Another study indicated that PCOS was positively correlated with an increased risk of developing breast cancer and, in particular, estrogen receptor (ER) positive breast cancer (17). The association between PCOS and endometrial cancer has, however, not yet been established through MR analysis.

In the present study, we sought to employ information from recent GWAS data on PCOS and endometrial cancer using a two-sample MR method to examine their causal relationship.

## Materials And Methods

### Genetic Instrumental Variables for PCOS

SNPs related to PCOS in Europeans were obtained from a GWAS meta-analysis that included 10,074 patients with PCOS and 103,164 health controls of European ancestry (18). In total, fourteen independent single nucleotide polymorphisms (SNPs) were estimated to be corelated to PCOS at the genome-wide significance level (P < 5×10^-8^). Of these SNPs, none were correlated (r^2^ < 0.001) in linkage disequilibrium (LD) analysis; one palindromic variant (rs853854) was excluded because of an effect allele frequency (EAF) close to 50% (19). The F-statistic was calculated to avoid weak IV bias (20, 21), where IVs with F-statistics > 10 were considered as strong IVs. In the present study, all IVs were strong IVs; therefore, thirteen SNPs in total were included to construct the genetic IVs for PCOS in Europeans (**Table 1**).

**Table 1:** PCOS SNPs used to construct the instrument variable in Europeans.

Chr	Position	SNP	Effect Allele	Other Allele	EAF	Beta	SE	Gene	P value
2	43561780	rs7563201	A	G	0.4507	-0.1081	0.0172	THADA	3.68E-10
2	213391766	rs2178575	A	G	0.1512	0.1663	0.0219	ERBB4	3.34E-14
3	131813204	rs13164856	T	C	0.7291	0.1235	0.0193	IRF1/RAD50	1.45E-10
8	11623889	rs804279	A	T	0.2616	0.1276	0.0184	GATA4/NEIL2	3.76E-12
9	5440589	rs10739076	A	C	0.3078	0.1097	0.0197	PLGRKT	2.51E-08
9	97723266	rs7864171	A	G	0.4284	-0.0933	0.0168	FANCC	2.95E-08
9	126619233	rs9696009	A	G	0.0679	0.202	0.0311	DENND1A	7.96E-11
11	30226356	rs11031005	T	C	0.8537	-0.1593	0.0223	ARL14EP/FSHB	8.66E-13
11	102043240	rs11225154	A	G	0.0941	0.1787	0.0272	YAP1	5.44E-11
11	113949232	rs1784692	T	C	0.8237	0.1438	0.0226	ZBTB16	1.88E-10
12	56477694	rs2271194	A	T	0.416	0.0971	0.0166	ERBB3/RAB5B	4.57E-09
12	75941042	rs1795379	T	C	0.2398	-0.1174	0.0195	KRR1	1.81E-09
16	52375777	rs8043701	A	T	0.815	-0.1273	0.0208	TOX3	9.61E-10

Chr, chromosome; SNP, single nucleotide polymorphism; EAF, effect allele frequency; SE, standard error.

We identified SNPs that were significantly related to PCOS in Asians from two PCOS GWAS datasets on cohorts of Han Chinese ancestry (22, 23). A GWAS consisting of 4082 PCOS cases and 6687 controls identified three independent SNPs that were strongly associated with PCOS (22). Another GWAS, including 10,480 cases and 10,579 controls, discovered ten novel PCOS-associated SNPs (23). Eventually, thirteen independent SNPs were adopted as IVs for PCOS in Asians after checking for LD (r^2^ < 0.001), palindromic SNP and weak IVs (**Table 2**).

**Table 2:** PCOS SNPs used to construct the instrument variable in Asians.

Chr	Position	SNP	Effect Allele	Other Allele	EAF	Beta	SE	Gene	P value
2	43638838	rs13429458	A	C	0.81	0.401	0.04	THADA	1.73E-23
2	48978159	rs13405728	A	G	0.754	0.343	0.037	LHCGR	7.55E-21
2	49201612	rs2268361	C	T	0.504	0.139	0.02	FSHR	9.89E-13
2	49247832	rs2349415	T	C	0.181	0.174	0.025	FSHR	2.35E-12
9	97648587	rs4385527	G	A	0.781	0.174	0.03	C9orf3(AOPEP)	5.87E-09
9	97741336	rs3802457	G	A	0.904	0.261	0.035	C9orf3(AOPEP)	5.28E-14
9	126525212	rs2479106	G	A	0.222	0.293	0.033	DENND1A	8.12E-19
11	102070639	rs1894116	G	A	0.194	0.239	0.024	YAP1	1.08E-22
12	56390636	rs705702	G	A	0.245	0.239	0.023	RAB5B/SUOX	8.64E-26
12	66224461	rs2272046	A	C	0.907	0.357	0.038	HMGA2	1.95E-21
16	52347819	rs4784165	G	T	0.325	0.14	0.021	TOX3	3.64E-11
19	7166109	rs2059807	G	A	0.301	0.131	0.023	INSR	1.09E-08
20	52447303	rs6022786	A	G	0.339	0.122	0.02	SUMO1P1	1.83E-09

Chr, chromosome; SNP, single nucleotide polymorphism; EAF, effect allele frequency; SE, standard error.

### GWAS on Endometrial Cancer

Genetic association data on endometrial cancer in Europeans was acquired from the largest published meta-GWAS of endometrial cancer to date, which includes a total of 12,906 endometrial cancer patients and 108,979 country-matched health participants of European ancestry from seventeen studies identified *via* the UK Biobank, the Endometrial Cancer Association Consortium (ECAC) and the Epidemiology of Endometrial Cancer Consortium (E2C2) (24). These endometrial cancer cases were further divided into an endometrioid histology group (8758 cases) and a non-endometrioid histology group (with serous carcinoma, carcinosarcoma, clear cell carcinoma or mucinous carcinoma) (1230 cases) according to the histological subtypes of endometrial cancer (24, 25). In this work, we extracted overall and histotype-specific endometrial cancer–specific beta coefficients and standard errors from the summary statistics of the meta-GWAS for each of the 13 SNPs for PCOS in Europeans.

Genetic association data on endometrial cancer in Asians were acquired from the BioBank Japan Project (BBJ), which includes 999 cases and 89,731 controls of Asian ancestry (26). BBJ recruited participants from 12 cooperating medical institutions in Japan. The identification of endometrial cancer cases was based on diagnoses by physicians at each hospital. Disease-specific laboratory examinations and imaging data were collected in BBJ (27), but these data did not include information about the histology of the endometrial cancer. Similarly, we extracted overall endometrial cancer–specific beta coefficients and standard errors from the GWAS summary level results for each of the 13 SNPs for PCOS in Asians.

### Statistical Analysis

MR is a statistical method of using genetic variants related to a modifiable exposure to examine whether an observational effect between this specific exposure and the outcome is consistent with a causal association (14). To obtain a reliable understanding for MR analysis, three prerequisite assumptions must be satisfied (28): (a) the IVs are strongly associated with PCOS; (b) the IVs can only affect endometrial cancer through their effects on PCOS; (c) the IVs are independent of any confounding factors that may influence the association between PCOS and endometrial cancer. In this case, MR analysis is less susceptible to reverse causation and confounding factors, which may greatly influence the results of epidemiological observational studies.

We evaluated the association of PCOS with overall and histotype-specific endometrial cancer risk. MR analyses in Europeans and Asians were conducted using an IV consisting of 13 SNPs for PCOS in Europeans and a 13-SNP IV for PCOS in Asians, respectively. The inverse-variance weighted (IVW) method was carried out as a main method of MR analysis. Odds ratios (OR) and 95% confidence intervals (CIs) for overall endometrial cancer risk, as well as both subtypes of endometrial cancer risk, were estimated.

Regarding sensitivity analyses, MR-Egger regression, weighted median, simple mode and weighted mode methods were employed in our study to assess whether the IVs could affect endometrial cancer merely through their impact on PCOS. MR-Egger regression, a method for detecting small study reporting bias in meta-analysis, was adapted to assess bias from pleiotropic effects. As such, the beta coefficient from an Egger regression can provide a consistent estimate of any causal effect (29). The weighted median method is able to provide a consistent estimate of the finding if more than half of the weight is derived from valid IVs (30). The simple mode method can also provide a consistent estimate if the most common horizontal pleiotropy value is zero, regardless of the type of horizontal pleiotropy. Also, the weighted mode method requires that the largest subset of instruments that demonstrates the same association is contributed by valid IVs (31). We also applied the MR-Pleiotropy Residual Sum and Outlier (MR-PRESSO) method to detect and correct horizontal pleiotropy and potential outliers (32). Finally, the heterogeneity of the association was also tested using Cochran’s Q test on the IVW and MR-Egger estimates.

We also carried out additional sensitivity analyses taking the potential confounders of endometrial cancer into account. It is well known that endometrial cancer has a strong association with obesity, high BMI and waist-to-hip ratio (WHR) (10, 33), all of which may increase the risk of PCOS (6, 34). Oral contraceptives (OCs), which have been widely accepted as a first line of treatment against PCOS, have also been found to be linked to a reduced risk of endometrial cancer (35). Additionally, parity may also be a potential confounding factor in this study because, on the one hand, it is known that PCOS is a major cause of female infertility (36) and, on the other hand, decreased parity is also shown to be associated with an increased risk of endometrial cancer (33).

We assessed whether PCOS‐associated SNPs were correlated with the aforementioned potential confounders at a genome-wide significance level (P < 5.0×10^-8^) by searching through the PhenoScanner database (http://www.phenoscanner.medschl.cam.ac.uk) (37). Among individuals of European ancestry, we found that rs2271194 was significantly associated with BMI, whereas in Asian PCOS SNPs, we found that rs705702 was correlated with BMI. We also examined whether PCOS SNPs were in LD (r^2^ > 0.2) with genome-wide significant signals for the following confounding factors: BMI, WHR, OC use and parity. Since there is no GWAS assessing OC use, we did not assess the association of included PCOS SNPs with OC and parity. Two European PCOS SNPs (rs9696009 and rs2271194) and two Asian PCOS SNPs (rs2479106 and rs705702) are linked to SNPs for increases in BMI, and one European PCOS SNP (rs7563201) and three Asian PCOS SNPs (rs13429458, rs2059807 and rs6022786) are linked to SNPs for increases in WHR (see **Supplementary Table 1**).

Thus, we conducted a series of sensitivity analyses in which a subset of PCOS SNPs, excluding SNPs associated with confounders, was used as IVs. In Europeans, we used the following groups of IVs: (a) eleven SNPs after removing two SNPs linked to BMI; (b) twelve SNPs after dismissing one SNP linked to WHR; (c) ten SNPs after discharging all three of these SNPs. In Asians, we used the following groups of IVs: (a) eleven SNPs after removing two SNPs linked to BMI; (b) ten SNPs after dismissing three SNPs linked to WHR; (c) eight SNPs after discharging all five of these SNPs. MR analyses and sensitivity analyses were performed in R (version 4.0.2) using the TwoSampleMR package (version 0.5.5) and the MRPRESSO package (version 1.0).

## Results

**Figure 1:**
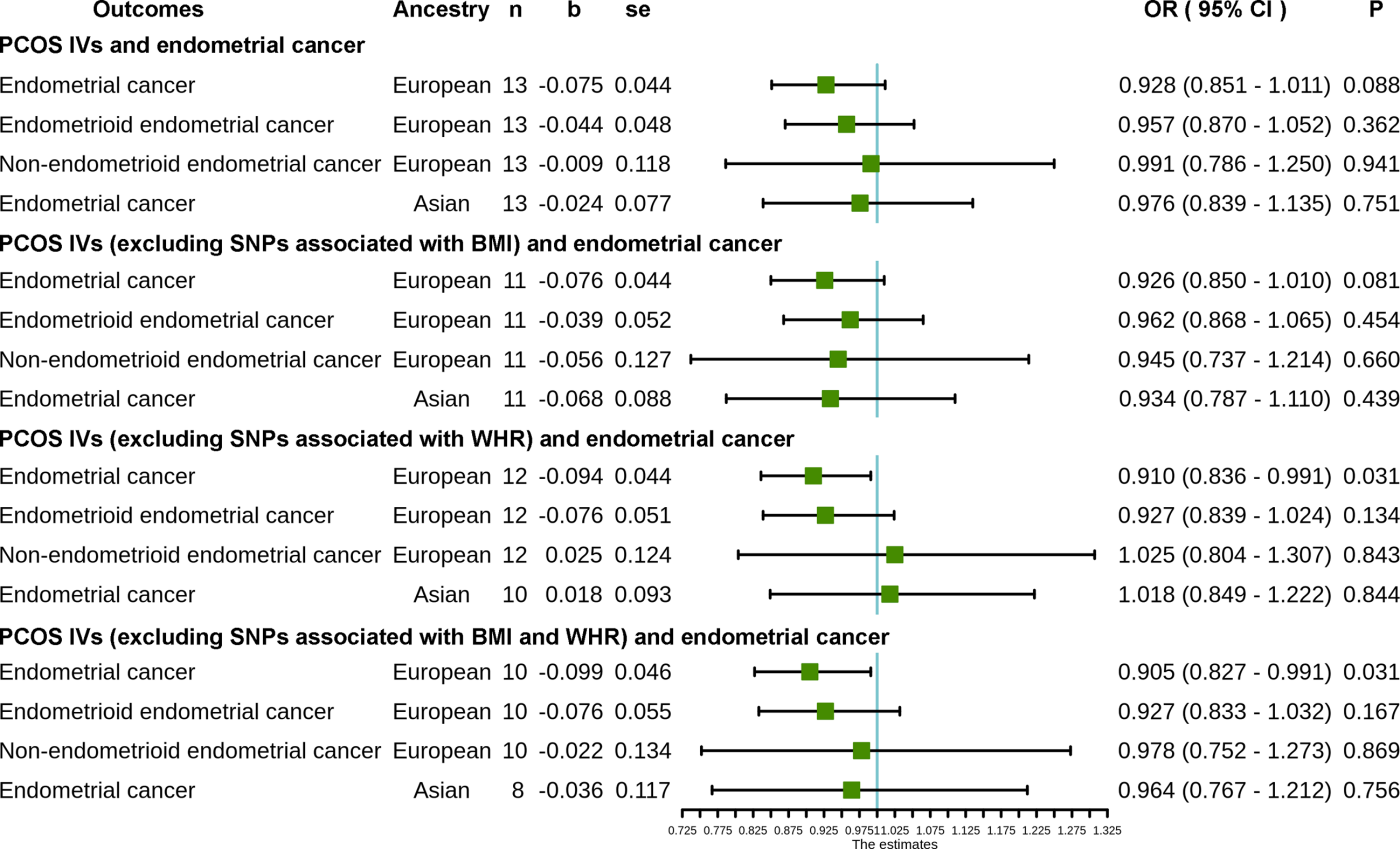
Causal effect estimates of PCOS on endometrial cancer: the inverse-variance weighted (IVW) method was applied as the primary method for MR analysis. Abbreviations: PCOS, polycystic ovary syndrome; IVS, instrumental variables; SNP, single nucleotide polymorphism; n, number (number of SNPs included in the analysis); b, beta coefficient; se, standard error; OR, odds ratio; CI, confidence interval; BMI, body mass index; WHR, waist-to-hip ratio.

The causal effect estimates of PCOS on endometrial cancer are displayed in **Figure 1** and **Supplementary Figure 1**. In this study, we did not observe a significant association between genetically predicted PCOS and the risk of endometrial cancer in either European ancestry or Asian ancestry (OR = 0.93, 95% CI 0.85–1.01, p = 0.09 and OR = 0.98, 95% CI 0.84–1.13, p = 0.75, respectively). Subgroup analyses according to histotype indicate that PCOS is not significantly associated with the risk of either endometrioid endometrial cancer (OR = 0.96, 95% CI 0.87–1.05, p = 0.36) or non-endometrioid endometrial cancer (OR = 0.99, 95% CI 0.79–1.25, p = 0.94) in European ancestry.

In all cases, the MR-Egger and MR-PRESSO results are not statistically significant (p > 0.05), demonstrating an absence of directional pleiotropy (**Table 3**). In sensitivity analyses using the weighted median, simple mode and weighted mode methods, the results are similar with those of IVW (**Table 3**). In addition, we did not detect substantial heterogeneity in any of our results.

**Table 3:** Associations between genetically predicted PCOS and endometrial cancer in Asians and Europeans using MR-Egger, weighted median, inverse variance weighted, simple mode, weighted mode and MR-PRESSO methods.

Outcomes	Number of SNPs	Beta	SE	OR (95% CI)	P	P for heterogeneity test	P for MR-Egger intercept	P for MR-PRESSO Global test
**Endometrial Cancer in Europeans**								
MR Egger	13	-0.04	0.218	0.961 (0.627 - 1.472)	0.857	0.236	0.873	
Weighted median	13	-0.043	0.062	0.958 (0.849 - 1.081)	0.489			
Inverse variance weighted	13	-0.075	0.044	0.928 (0.851 - 1.011)	0.088	0.302		
Simple mode	13	-0.01	0.109	0.990 (0.799 - 1.227)	0.928			
Weighted mode	13	-0.029	0.115	0.971 (0.775 - 1.217)	0.805			
MR-PRESSO (raw, 0 outliers)	13	-0.058	0.045	0.944 (0.864 - 1.031)	0.222			0.190
**Endometrioid Endometrial Cancer in Europeans**								
MR Egger	13	-0.12	0.23	0.887 (0.564 - 1.393)	0.612	0.441	0.742	
Weighted median	13	-0.033	0.07	0.967 (0.843 - 1.109)	0.632			
Inverse variance weighted	13	-0.044	0.048	0.957 (0.870 - 1.052)	0.362	0.517		
Simple mode	13	-0.022	0.117	0.978 (0.778 - 1.230)	0.854			
Weighted mode	13	-0.029	0.111	0.972 (0.781 - 1.208)	0.800			
MR-PRESSO (raw, 0 outliers)	13	-0.018	0.051	0.982 (0.889 - 1.085)	0.729			0.269
**Non-Endometrioid Endometrial Cancer in Europeans**								
MR Egger	13	0.521	0.565	1.684 (0.557 - 5.095)	0.376	0.449	0.358	
Weighted median	13	0	0.162	1.000 (0.728 - 1.375)	0.999			
Inverse variance weighted	13	-0.009	0.118	0.991 (0.786 - 1.250)	0.941	0.457		
Simple mode	13	0.041	0.285	1.042 (0.596 - 1.820)	0.889			
Weighted mode	13	0.035	0.292	1.036 (0.584 - 1.836)	0.906			
MR-PRESSO (raw, 0 outliers)	13	-0.004	0.109	0.996 (0.804 - 1.233)	0.973			0.505
**Endometrial Cancer in Asians**								
MR Egger	13	0.01	0.204	1.010 (0.678 - 1.505)	0.963	0.091	0.858	
Weighted median	13	-0.011	0.092	0.989 (0.826 - 1.185)	0.907			
Inverse variance weighted	13	-0.024	0.077	0.976 (0.839 - 1.135)	0.751	0.127		
Simple mode	13	-0.022	0.158	0.978 (0.717 - 1.335)	0.893			
Weighted mode	13	-0.002	0.112	0.998 (0.801 - 1.242)	0.983			
MR-PRESSO (raw, 0 outliers)	13	-0.0245	0.077	0.976 (0.839 - 1.135)	0.756			0.132

SNP, single nucleotide polymorphism; SE, standard error; OR, odds ratio; CI, confidential interval.

Additional sensitivity analyses also show results similar to our primary findings. We did not observe a statistically significant association between IVs composed of PCOS SNPs not associated with BMI and endometrial cancer in either Europeans or Asians (see **Supplementary Table 2**).

After excluding PCOS SNPs associated with WHR, however, our results show that genetically predicted PCOS is correlated to a reduced risk of overall endometrial cancer in European ancestry (OR = 0.91, 95% CI 0.84–0.99, p = 0.03). This association is absent in Asians and subgroup analyses according to histotype in Europeans (see **Supplementary Table 3**). Furthermore, PCOS is also associated with a reduced risk of overall endometrial cancer in Europeans after removing SNPs related to BMI or WHR (OR = 0.91, 95% CI 0.83–0.99, p = 0.03). Still, this association is absent in Asians and subgroup analyses in Europeans (**Supplementary Table 4**).

## Discussion

For the first time, our MR study evaluates the association between PCOS and endometrial cancer, and our findings suggest that PCOS is not causally related to the risk of endometrial cancer in either European or Asian ancestry.

Previously, numerous observational studies found controversial conclusions on whether the risk of endometrial cancer is associated with PCOS. One meta-analysis demonstrated that PCOS was associated with a higher risk of endometrial cancer in women with an assessed OR of 2.79 (95% CI 1.31–5.95) (38). All of the included studies, however, were of moderate or low quality because none of them adjusted their results for confounding factors, such as BMI, diabetes and inflammation. One study found that women with PCOS have a higher risk of endometrial cancer compared to their age-matched controls, regardless of BMI (OR _unadjusted_  = 5.3, 95% CI 1.5–18.6 and OR _adjusted_  = 6.1, 95% CI 1.0–36.9, respectively) (39). On the contrary, two studies found no relationship between PCOS and risk of endometrial cancer after adjusting for BMI (OR _BMI-adjusted_  = 2.2, 95% CI 0.9–5.7 and OR _BMI-adjusted_ = 1.3, 95% CI 0.7–2.2, respectively) (40, 41). The contradictory conclusions of these observational studies might have been caused by confounding factors. Thus, a major advantage of our study is that MR can control for the influence of confounding factors. Moreover, endometrial cancer has two main subtypes: endometrioid (Type I) and non-endometrioid (Type II). But only one study investigated the association between PCOS and the subtypes of endometrial cancer, reporting a slightly stronger relationship between PCOS and Type I endometrial cancer (OR _endometrioid_  = 2.4, 95% CI 1.0–6.2) (41).

In our study, we did not find a significant association between PCOS and the risk of endometrial cancer. After removing SNPs associated with potential confounders and subgroup analyses according to histotype, our MR study concluded that PCOS still does not have any causal association with the risk of endometrial cancer. Although our results indicate that PCOS does not directly increase the risk of endometrial cancer, the obesity feature of PCOS has been found to be carcinogenic in numerous observational and MR studies (33, 42). The findings of increased BMI in PCOS women might explain the association between PCOS and the increased risk of endometrial cancer, especially in studies with no adjustment for BMI.

Our study contains several advantages. First, a major strength of this study is its two-sample MR design, which can prevent the influence of reverse causality and potential confounding factors. Second, the study’s IVs are derived from the latest and largest PCOS GWAS in Asians and Europeans (18, 22, 23), and our endometrial cancer data were obtained from the latest and biggest endometrial cancer GWAS in both ethnic groups (24, 26), which can thus better represent exposure and outcomes. Therefore, our study can provide sufficient statistical strength and precise estimates of causal effects. Third, we conducted sensitivity analyses for confounding factors and pleiotropy. Using MR Egger and MR-PRESSO analyses, we did not detect horizontal pleiotropy. In addition, we also assessed the impact of potential confounding factors, such as BMI and WHR (10). After removing SNPs associated with these potential confounders, our MR study suggests that genetically predicted PCOS still does not have any causal association with the risk of endometrial cancer, suggesting that there is no independent association between PCOS and endometrial cancer. Moreover, we stratified our outcomes based on the histotype of endometrial cancer, which was often neglected by previous observation studies. In our study, the inverse relationship between PCOS and overall endometrial cancer in Europeans after removing SNPs associated with (a) WHR and (b) WHR or BMI is diminished by subgroup analysis according to histotype, further revealing the negative association between PCOS and endometrial cancer.

Nonetheless, our study still has several limitations. First, we found that PCOS is not related to endometrial cancer in two ethnic groups based on GWAS data summaries of European and Asian populations. Therefore, it is unclear whether our results are still applicable to other populations. Also, for the analysis in Asians, the selected IVs were based on a Chinese PCOS GWAS, and the outcomes were from a GWAS of Japanese endometrial cancer cases and controls. These two east Asian populations are not genetically identical, but they are genetically closely related and comparable. Studies have reported that the Han Chinese and Japanese ethnic groups show similar patterns for most genetic polymorphisms (43). But some researchers have also found that they are less similar regarding genome-wide variations (44), which may have influenced the accuracy of our study. Second, we did not assess the causal relationship between body weight, BMI, WHR and PCOS, as well as their causal association with endometrial cancer, because previous studies have already investigated these aspects and found that an increase in BMI is causally associated with PCOS and endometrial cancer risk (6, 42, 45, 46). Third, due to limited information, we were unable to verify that the PCOS-related SNPs we selected are related to individuals known for having PCOS in their outcome (i.e. endometrial cancer) databases. The lack of this control experiment decreases the rationality of our study because the association between genetics and clinics cannot be fully verified. Fourth, our study was not able to assess the association between each PCOS phenotype and the risk of endometrial cancer. In our study, the Asian GWAS for PCOS used Rotterdam criteria, whereas the European PCOS GWAS utilized the NIH/NICHD criteria (14.6%), self-reported diagnosis (51.4%) and also the Rotterdam criteria (34.0%) (18). This might affect the specificity of the European PCOS IVs. In 2012, the NIH consensus panel recommended the following clinical phenotype classification for PCOS: (a) Phenotype A consists of ovulatory dysfunction (OD), hyperandrogenism (HA) (clinical or biochemical) and polycystic ovaries (PCO); (b) Phenotype B consists of HA and OD; (c) Phenotype C consists of PCO and HA; (d) Phenotype D consists of PCO and OD (47). We failed, however, to stratify our outcomes according to the different clinical phenotypes of PCOS due to a lack of information and since we were not able to identify the risks of each phenotype of PCOS, which is quite significant in clinical practice. Further studies using different clinical phenotypes of PCOS are needed to fully explore the association between PCOS and endometrial cancer.

## Conclusions

In conclusion, based on the MR results generated using data summaries from large-scale GWAS analyses, our observations suggest that genetically predicted PCOS is not related to a higher risk of endometrial cancer.

## Data Availability Statement

Publicly available datasets were analyzed in this study. This data can be found here: https://www.repository.cam.ac.uk/handle/1810/289950, https://gwas.mrcieu.ac.uk/datasets/ and http://jenger.riken.jp/en/result.

## Author Contributions

Conceptualization: LQ, YW, HC, and YZ. Data curation and formal analysis: RG and KC. Funding acquisition: LQ. Software and visualization: HC, YZ, and YT. Supervision: SL, WX, and YY. Writing of the original draft: HC and YZ. Review and editing: LQ and YW. HC and YZ verified the underlying data. All authors contributed to the article and approved the submitted version.

## Funding

This work was supported by Sichuan Science and Technology Program [2020YFS0127].

## Conflict of Interest

The authors declare that the research was conducted in the absence of any commercial or financial relationships that could be construed as a potential conflict of interest.

## Publisher’s Note

All claims expressed in this article are solely those of the authors and do not necessarily represent those of their affiliated organizations, or those of the publisher, the editors and the reviewers. Any product that may be evaluated in this article, or claim that may be made by its manufacturer, is not guaranteed or endorsed by the publisher.

## Supplementary Material

Supplementary Figure 1 Scatter plot of PCOS on endometrial cancer: **(A)** scatter plot of PCOS on endometrial cancer in Asians; **(B)** scatter plot of PCOS on overall endometrial cancer in Europeans; **(C)** scatter plot of PCOS on endometrioid endometrial cancer in Europeans; **(D)** scatter plot of PCOS on non-endometrioid endometrial cancer in Europeans.
