# Supplementary material for: The Genetic Association of Polycystic Ovary Syndrome and the Risk of Endometrial Cancer: A Mendelian Randomization Study
Source: Front Endocrinol (Lausanne). 2021 Nov 5;12:756137. doi: 10.3389/fendo.2021.756137 (PMC8602912; doi:10.3389/fendo.2021.756137)
Supplement: Supplementary Figure 1 — Scatter plot of PCOS on endometrial cancer: (A) scatter plot of PCOS on endometrial cancer in Asians; (B) scatter plot of PCOS on overall endometrial cancer in Europeans; (C) scatter plot of PCOS on endometrioid endometrial cancer in Europeans; (D) scatter plot of PCOS on non-endometrioid endometrial cancer in Europeans. [file DataSheet_1.zip › supplementary meterials/Supplemental Table 1.docx]

**Supplemental Table 1. Linkage disequilibrium between PCOS SNPs and genome-wide significant signals for BMI and WHR.**

| **Ancestry** | **Position** | **PCOS SNP** | **Nearest Gene** | **Related Traits** | **Linked SNP** | **Position** | **r2** | **Beta or OR** | **P value** | **GWAS catlog** |
| --- | --- | --- | --- | --- | --- | --- | --- | --- | --- | --- |
| European | 2:43561780 | rs7563201 | THADA | Waist-to-hip ratio adjusted for BMI | rs11124930 | chr2:43594489 | 0.262 | NA | 4x10-8 | <https://www.ebi.ac.uk/gwas/variants/rs11124930> |
|  |  |  |  | Waist-to-hip ratio adjusted for BMI | rs11694173 | chr2:43590899 | 0.217 | 0.014 | 3x10-11 | <https://www.ebi.ac.uk/gwas/variants/rs11694173> |
|  |  |  |  | Waist-hip ratio | rs11694173 | chr2:43590899 | 0.217 | 0.011 | 1x10-7 | <https://www.ebi.ac.uk/gwas/variants/rs11694173> |
| European | 9:126619233 | rs9696009 | DENND1A | Body mass index | rs541160 | chr9:126590687 | 0.376 | 0.023 | 1x10-9 | https://www.ebi.ac.uk/gwas/variants/rs541160 |
|  |  |  |  | Body mass index in non-asthmatics | rs10818854 | chr9:126446778 | 0.368 | NA | 2x10-7 | https://www.ebi.ac.uk/gwas/variants/rs10818854 |
| European | 12:56477694 | rs2271194 | ERBB3/RAB5B | Body mass index | rs705704 | chr12:56435412 | 0.643 | 0.014 | 6x10-15 | https://www.ebi.ac.uk/gwas/variants/rs705704 |
|  |  |  |  | Body mass index | rs4759228 | chr12:56508409 | 0.409 | NA | 1x10-16 | https://www.ebi.ac.uk/gwas/variants/rs4759228 |
|  |  |  |  | Body mass index | rs3759094 | chr12:56497903 | 0.387 | NA | 9x10-16 | https://www.ebi.ac.uk/gwas/variants/rs3759094 |
|  |  |  |  | Body mass index | rs10783779 | chr12:56491880 | 0.255 | 0.014 | 5x10-15 | https://www.ebi.ac.uk/gwas/variants/rs10783779 |
| Asian | 2:43638838 | rs13429458 | THADA | Waist-to-hip ratio adjusted for BMI | rs11124930 | chr2:43594489 | 0.538 | NA | 4x10-8 | https://www.ebi.ac.uk/gwas/variants/rs11124930 |
| Asian | 9:126525212 | rs2479106 | DENND1A | Body mass index | rs541160 | chr9:126590687 | 0.395 | 0.023 | 1x10-9 | https://www.ebi.ac.uk/gwas/variants/rs541160 |
| Asian | 12:56390636 | rs705702 | RAB5B/SUOX | Body mass index | rs705704 | chr12:56435412 | 0.777 | 0.014 | 6x10-15 | https://www.ebi.ac.uk/gwas/variants/rs705704 |
| Asian | 19:7166109 | rs2059807 | INSR | Waist-to-hip ratio adjusted for BMI | rs34194998 | chr19:7187860 | 0.227 | NA | 2x10-8 | https://www.ebi.ac.uk/gwas/variants/rs34194998 |
| Asian | 20:52447303 | rs6022786 | SUMO1P1 | Waist-hip ratio | rs6097585 | chr20:52421031 | 0.387 | 0.041 | 2x10-6 | https://www.ebi.ac.uk/gwas/variants/rs6097585 |
|  |  |  |  | Waist-to-hip ratio adjusted for BMI | rs6097585 | chr20:52421031 | 0.387 | 0.038 | 6x10-6 | https://www.ebi.ac.uk/gwas/variants/rs6097585 |
