# Supplementary material for: The Genetic Association of Polycystic Ovary Syndrome and the Risk of Endometrial Cancer: A Mendelian Randomization Study
Source: Front Endocrinol (Lausanne). 2021 Nov 5;12:756137. doi: 10.3389/fendo.2021.756137 (PMC8602912; doi:10.3389/fendo.2021.756137)
Supplement: Supplementary Figure 1 — Scatter plot of PCOS on endometrial cancer: (A) scatter plot of PCOS on endometrial cancer in Asians; (B) scatter plot of PCOS on overall endometrial cancer in Europeans; (C) scatter plot of PCOS on endometrioid endometrial cancer in Europeans; (D) scatter plot of PCOS on non-endometrioid endometrial cancer in Europeans. [file DataSheet_1.zip › supplementary meterials/supplemental table 2 PCOS_remove_BMI_res.csv.docx]

**Supplemental Table 2 The associations between PCOS (excluding SNPs associated with BMI) and endometrial cancer.**

| **Outcomes** | | **Number of SNPs** | **Beta** | **SE** | **OR (95% CI)** | **P** | **P for heterogeneity test** | **P for MR-Egger intercept** | **P for MR-PRESSO**  **Global test** |
| --- | --- | --- | --- | --- | --- | --- | --- | --- | --- |
| **Endometrial Cancer in Europeans** | |  |  |  |  |  |  |  |  |
|  | MR Egger | 11 | -0.376 | 0.241 | 0.687 (0.428 - 1.101) | 0.153 | 0.501 | 0.237 |  |
|  | Weighted median | 11 | -0.046 | 0.059 | 0.955 (0.85 - 1.072) | 0.434 |  |  |  |
|  | Inverse variance weighted | 11 | -0.076 | 0.044 | 0.926 (0.85 - 1.01) | 0.081 | 0.446 |  |  |
|  | Simple mode | 11 | -0.028 | 0.101 | 0.972 (0.798 - 1.184) | 0.785 |  |  |  |
|  | Weighted mode | 11 | -0.039 | 0.107 | 0.962 (0.779 - 1.187) | 0.723 |  |  |  |
|  | MR-PRESSO (raw, 0 outliers) | 11 | -0.056 | 0.046 | 0.945 (0.864 - 1.033) | 0.241 |  |  | 0.338 |
| **Endometrioid Endometrial Cancer in Europeans** | |  |  |  |  |  |  |  |  |
|  | MR Egger | 11 | -0.390 | 0.287 | 0.677 (0.386 - 1.187) | 0.206 | 0.511 | 0.244 |  |
|  | Weighted median | 11 | -0.034 | 0.071 | 0.967 (0.84 - 1.112) | 0.636 |  |  |  |
|  | Inverse variance weighted | 11 | -0.039 | 0.052 | 0.962 (0.868 - 1.065) | 0.454 | 0.459 |  |  |
|  | Simple mode | 11 | -0.036 | 0.118 | 0.965 (0.765 - 1.216) | 0.766 |  |  |  |
|  | Weighted mode | 11 | -0.043 | 0.114 | 0.958 (0.765 - 1.199) | 0.714 |  |  |  |
|  | MR-PRESSO (raw, 0 outliers) | 11 | -0.01 | 0.057 | 0.99 (0.886 - 1.107) | 0.868 |  |  | 0.217 |
| **Non-Endometrioid Endometrial Cancer in Europeans** | |  |  |  |  |  |  |  |  |
|  | MR Egger | 11 | -0.166 | 0.708 | 0.847 (0.211 - 3.395) | 0.820 | 0.538 | 0.878 |  |
|  | Weighted median | 11 | -0.001 | 0.174 | 0.999 (0.71 - 1.404) | 0.993 |  |  |  |
|  | Inverse variance weighted | 11 | -0.056 | 0.127 | 0.945 (0.737 - 1.214) | 0.660 | 0.630 |  |  |
|  | Simple mode | 11 | 0.078 | 0.278 | 1.082 (0.628 - 1.863) | 0.783 |  |  |  |
|  | Weighted mode | 11 | 0.065 | 0.283 | 1.067 (0.613 - 1.857) | 0.823 |  |  |  |
|  | MR-PRESSO (raw, 0 outliers) | 11 | -0.047 | 0.105 | 0.954 (0.777 - 1.172) | 0.663 |  |  | 0.701 |
| **Endometrial Cancer in Asians** | |  |  |  |  |  |  |  |  |
|  | MR Egger | 11 | -0.048 | 0.216 | 0.953 (0.624 - 1.457) | 0.830 | 0.081 | 0.920 |  |
|  | Weighted median | 11 | -0.077 | 0.102 | 0.926 (0.759 - 1.131) | 0.452 |  |  |  |
|  | Inverse variance weighted | 11 | -0.068 | 0.088 | 0.934 (0.787 - 1.11) | 0.439 | 0.118 |  |  |
|  | Simple mode | 11 | -0.109 | 0.181 | 0.897 (0.628 - 1.279) | 0.560 |  |  |  |
|  | Weighted mode | 11 | -0.075 | 0.122 | 0.928 (0.73 - 1.179) | 0.554 |  |  |  |
|  | MR-PRESSO (raw, 0 outliers) | 11 | -0.068 | 0.088 | 0.934 (0.787 - 1.11) | 0.456 |  |  | 0.111 |

SNP, single nucleotide polymorphism; SE, standard error; OR, odds ratio; CI, confidential interval
