# Supplementary material for: The Genetic Association of Polycystic Ovary Syndrome and the Risk of Endometrial Cancer: A Mendelian Randomization Study
Source: Front Endocrinol (Lausanne). 2021 Nov 5;12:756137. doi: 10.3389/fendo.2021.756137 (PMC8602912; doi:10.3389/fendo.2021.756137)
Supplement: Supplementary Figure 1 — Scatter plot of PCOS on endometrial cancer: (A) scatter plot of PCOS on endometrial cancer in Asians; (B) scatter plot of PCOS on overall endometrial cancer in Europeans; (C) scatter plot of PCOS on endometrioid endometrial cancer in Europeans; (D) scatter plot of PCOS on non-endometrioid endometrial cancer in Europeans. [file DataSheet_1.zip › supplementary meterials/supplemental table 3 PCOS_remove_WHR_res.docx]

**Supplemental Table 3 The associations between PCOS (excluding SNPs associated with WHR) and endometrial cancer.**

| **Outcomes** | | **Number of SNPs** | **Beta** | **SE** | **OR (95% CI)** | **P** | **P for heterogeneity test** | **P for MR-Egger intercept** | **P for MR-PRESSO**  **Global test** |
| --- | --- | --- | --- | --- | --- | --- | --- | --- | --- |
| **Endometrial Cancer in Europeans** | |  |  |  |  |  |  |  |  |
|  | MR Egger | 12 | 0.034 | 0.209 | 1.034 (0.687 - 1.557) | 0.874 | 0.352 | 0.544 |  |
|  | Weighted median | 12 | -0.065 | 0.056 | 0.937 (0.839 - 1.045) | 0.241 |  |  |  |
|  | Inverse variance weighted | 12 | -0.094 | 0.044 | 0.91 (0.836 - 0.991) | 0.031 | 0.401 |  |  |
|  | Simple mode | 12 | -0.032 | 0.118 | 0.969 (0.769 - 1.221) | 0.794 |  |  |  |
|  | Weighted mode | 12 | -0.045 | 0.106 | 0.956 (0.778 - 1.176) | 0.680 |  |  |  |
|  | MR-PRESSO (raw, 0 outliers) | 12 | -0.074 | 0.046 | 0.929 (0.849 - 1.015) | 0.130 |  |  | 0.268 |
| **Endometrioid Endometrial Cancer in Europeans** | |  |  |  |  |  |  |  |  |
|  | MR Egger | 12 | -0.010 | 0.236 | 0.99 (0.624 - 1.573) | 0.968 | 0.780 | 0.780 |  |
|  | Weighted median | 12 | -0.036 | 0.066 | 0.964 (0.846 - 1.098) | 0.583 |  |  |  |
|  | Inverse variance weighted | 12 | -0.076 | 0.051 | 0.927 (0.839 - 1.024) | 0.134 | 0.838 |  |  |
|  | Simple mode | 12 | -0.022 | 0.119 | 0.979 (0.774 - 1.236) | 0.859 |  |  |  |
|  | Weighted mode | 12 | -0.029 | 0.104 | 0.972 (0.792 - 1.192) | 0.789 |  |  |  |
|  | MR-PRESSO (raw, 0 outliers) | 12 | -0.045 | 0.048 | 0.956 (0.87 - 1.05) | 0.363 |  |  | 0.485 |
| **Non-Endometrioid Endometrial Cancer in Europeans** | |  |  |  |  |  |  |  |  |
|  | MR Egger | 12 | 0.437 | 0.593 | 1.548 (0.484 - 4.951) | 0.478 | 0.398 | 0.493 |  |
|  | Weighted median | 12 | 0.028 | 0.171 | 1.029 (0.736 - 1.437) | 0.868 |  |  |  |
|  | Inverse variance weighted | 12 | 0.025 | 0.124 | 1.025 (0.804 - 1.307) | 0.843 | 0.441 |  |  |
|  | Simple mode | 12 | 0.069 | 0.290 | 1.072 (0.607 - 1.893) | 0.816 |  |  |  |
|  | Weighted mode | 12 | 0.064 | 0.295 | 1.066 (0.598 - 1.9) | 0.832 |  |  |  |
|  | MR-PRESSO (raw, 0 outliers) | 12 | 0.028 | 0.115 | 1.028 (0.821 - 1.287) | 0.814 |  |  | 0.510 |
| **Endometrial Cancer in Asians** | |  |  |  |  |  |  |  |  |
|  | MR Egger | 10 | 0.331 | 0.277 | 1.392 (0.809 - 2.396) | 0.266 | 0.189 | 0.266 |  |
|  | Weighted median | 10 | 0.092 | 0.105 | 1.096 (0.892 - 1.347) | 0.384 |  |  |  |
|  | Inverse variance weighted | 10 | 0.018 | 0.093 | 1.018 (0.849 - 1.222) | 0.844 | 0.153 |  |  |
|  | Simple mode | 10 | 0.038 | 0.152 | 1.039 (0.771 - 1.401) | 0.807 |  |  |  |
|  | Weighted mode | 10 | 0.105 | 0.111 | 1.111 (0.893 - 1.381) | 0.370 |  |  |  |
|  | MR-PRESSO (raw, 0 outliers) | 10 | 0.018 | 0.093 | 1.018 (0.849 - 1.222) | 0.848 |  |  | 0.182 |

SNP, single nucleotide polymorphism; SE, standard error; OR, odds ratio; CI, confidential interval
