# Supplementary material for: The Genetic Association of Polycystic Ovary Syndrome and the Risk of Endometrial Cancer: A Mendelian Randomization Study
Source: Front Endocrinol (Lausanne). 2021 Nov 5;12:756137. doi: 10.3389/fendo.2021.756137 (PMC8602912; doi:10.3389/fendo.2021.756137)
Supplement: Supplementary Figure 1 — Scatter plot of PCOS on endometrial cancer: (A) scatter plot of PCOS on endometrial cancer in Asians; (B) scatter plot of PCOS on overall endometrial cancer in Europeans; (C) scatter plot of PCOS on endometrioid endometrial cancer in Europeans; (D) scatter plot of PCOS on non-endometrioid endometrial cancer in Europeans. [file DataSheet_1.zip › supplementary meterials/supplemental table 4 PCOS_remove_ALL_res.docx]

**Supplemental Table 4 The associations between PCOS (excluding SNPs associated with BMI and WHR) and endometrial cancer.**

| **Outcomes** | | **Number of SNPs** | **Beta** | **SE** | **OR (95% CI)** | **P** | **P for heterogeneity test** | **P for MR-Egger intercept** | **P for MR-PRESSO**  **Global test** |
| --- | --- | --- | --- | --- | --- | --- | --- | --- | --- |
| **Endometrial Cancer in Europeans** | |  |  |  |  |  |  |  |  |
|  | MR Egger | 10 | -0.286 | 0.252 | 0.751 (0.459 - 1.231) | 0.289 | 0.555 | 0.473 |  |
|  | Weighted median | 10 | -0.069 | 0.060 | 0.933 (0.83 - 1.05) | 0.249 |  |  |  |
|  | Inverse variance weighted | 10 | -0.099 | 0.046 | 0.905 (0.827 - 0.991) | 0.031 | 0.595 |  |  |
|  | Simple mode | 10 | -0.037 | 0.106 | 0.964 (0.783 - 1.187) | 0.736 |  |  |  |
|  | Weighted mode | 10 | -0.044 | 0.101 | 0.957 (0.784 - 1.167) | 0.674 |  |  |  |
|  | MR-PRESSO (raw, 0 outliers) | 10 | -0.075 | 0.046 | 0.927 (0.848 - 1.015) | 0.131 |  |  | 0.388 |
| **Endometrioid Endometrial Cancer in Europeans** | |  |  |  |  |  |  |  |  |
|  | MR Egger | 10 | -0.232 | 0.300 | 0.793 (0.441 - 1.426) | 0.461 | 0.765 | 0.610 |  |
|  | Weighted median | 10 | -0.037 | 0.069 | 0.964 (0.842 - 1.103) | 0.592 |  |  |  |
|  | Inverse variance weighted | 10 | -0.076 | 0.055 | 0.927 (0.833 - 1.032) | 0.167 | 0.815 |  |  |
|  | Simple mode | 10 | -0.037 | 0.113 | 0.964 (0.772 - 1.204) | 0.752 |  |  |  |
|  | Weighted mode | 10 | -0.040 | 0.107 | 0.96 (0.778 - 1.185) | 0.716 |  |  |  |
|  | MR-PRESSO (raw, 0 outliers) | 10 | -0.04 | 0.053 | 0.96 (0.865 - 1.066) | 0.466 |  |  | 0.433 |
| **Non-Endometrioid Endometrial Cancer in Europeans** | |  |  |  |  |  |  |  |  |
|  | MR Egger | 10 | -0.361 | 0.740 | 0.697 (0.163 - 2.975) | 0.639 | 0.521 | 0.654 |  |
|  | Weighted median | 10 | 0.027 | 0.181 | 1.027 (0.72 - 1.465) | 0.883 |  |  |  |
|  | Inverse variance weighted | 10 | -0.022 | 0.134 | 0.978 (0.752 - 1.273) | 0.869 | 0.600 |  |  |
|  | Simple mode | 10 | 0.076 | 0.305 | 1.079 (0.593 - 1.963) | 0.810 |  |  |  |
|  | Weighted mode | 10 | 0.059 | 0.289 | 1.061 (0.602 - 1.871) | 0.842 |  |  |  |
|  | MR-PRESSO (raw, 0 outliers) | 10 | -0.015 | 0.111 | 0.985 (0.793 - 1.224) | 0.896 |  |  | 0.653 |
| **Endometrial Cancer in Asians** | |  |  |  |  |  |  |  |  |
|  | MR Egger | 8 | 0.245 | 0.324 | 1.278 (0.677 - 2.412) | 0.478 | 0.117 | 0.388 |  |
|  | Weighted median | 8 | 0.024 | 0.123 | 1.025 (0.805 - 1.303) | 0.844 |  |  |  |
|  | Inverse variance weighted | 8 | -0.036 | 0.117 | 0.964 (0.767 - 1.212) | 0.756 | 0.112 |  |  |
|  | Simple mode | 8 | -0.026 | 0.205 | 0.974 (0.652 - 1.455) | 0.901 |  |  |  |
|  | Weighted mode | 8 | 0.061 | 0.125 | 1.063 (0.833 - 1.358) | 0.638 |  |  |  |
|  | MR-PRESSO (raw, 0 outliers) | 8 | -0.036 | 0.117 | 0.964 (0.767 - 1.212) | 0.765 |  |  | 0.110 |

SNP, single nucleotide polymorphism; SE, standard error; OR, odds ratio; CI, confidential interval
