# Supplementary figures and images for: The Genetic Association of Polycystic Ovary Syndrome and the Risk of Endometrial Cancer: A Mendelian Randomization Study
Source: Front Endocrinol (Lausanne). 2021 Nov 5;12:756137. doi: 10.3389/fendo.2021.756137 (PMC8602912; doi:10.3389/fendo.2021.756137)

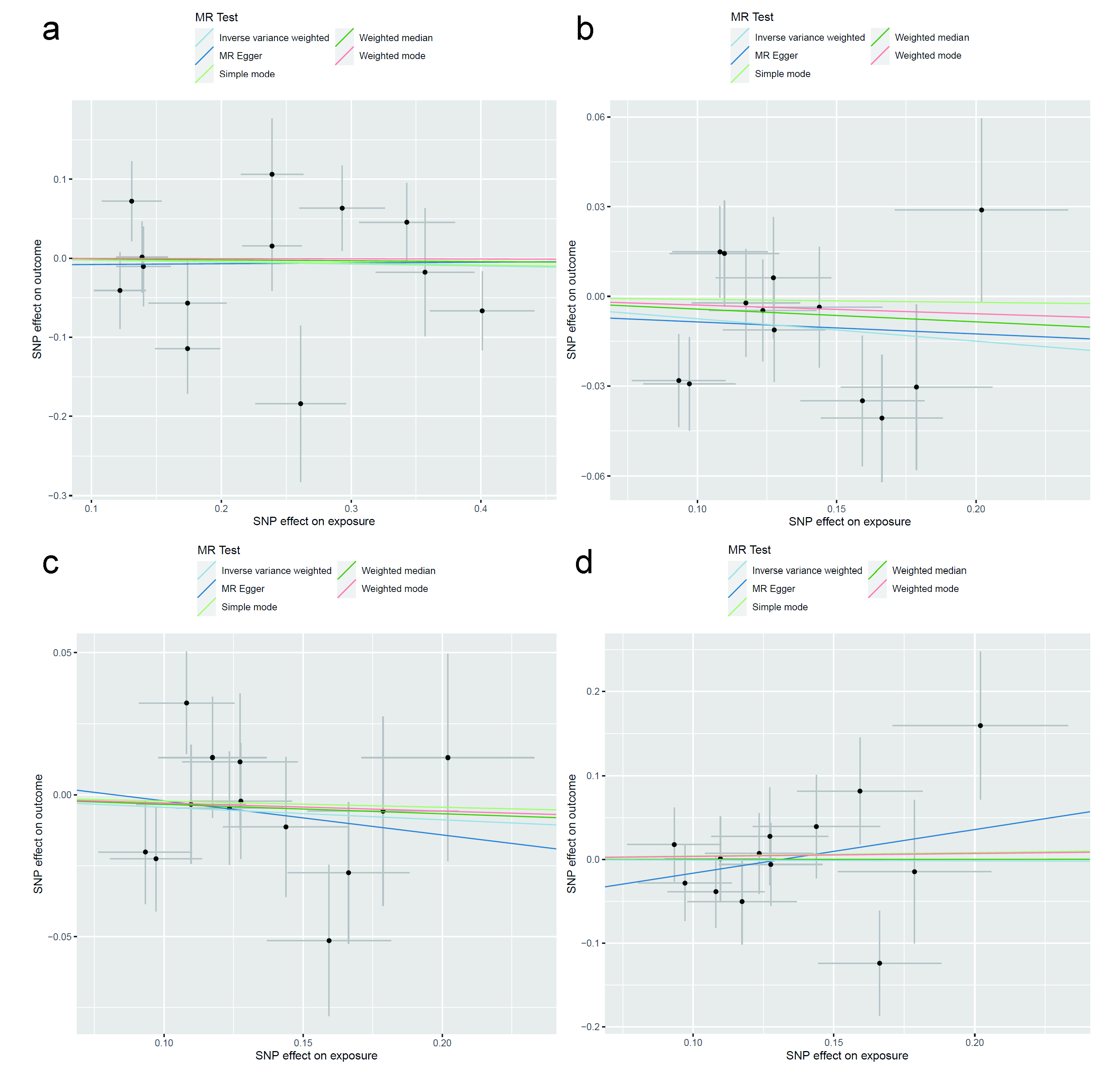

Supplement: Supplementary Figure 1 — Scatter plot of PCOS on endometrial cancer: (A) scatter plot of PCOS on endometrial cancer in Asians; (B) scatter plot of PCOS on overall endometrial cancer in Europeans; (C) scatter plot of PCOS on endometrioid endometrial cancer in Europeans; (D) scatter plot of PCOS on non-endometrioid endometrial cancer in Europeans. [file DataSheet_1.zip › supplementary meterials/supplemental figure 1 Scatter plot of PCOS on endometrial cancer.tiff]
